# 11384 Medication Use Safety During Care Transitions for Children with Medical Complexity

**DOI:** 10.1017/cts.2021.740

**Published:** 2021-03-30

**Authors:** Ephrem Abebe, Sarah Wiehe, Richard J. Holden

**Affiliations:** 1 Purdue University College of Pharmacy and Indiana University School of Medicine; 2 Indiana University, School of Medicine

## Abstract

ABSTRACT IMPACT: This study will generate preliminary data to address a critical, care transition-related patient safety gap involving medication use among children with medical complexity. OBJECTIVES/GOALS: The objectives of this study are: (1) to understand care transition-related medication safety risks for children with medical complexity (CMC), and (2) through a participatory, human centered design (PD) approach, to develop an early prototype intervention to address identified safety risks. METHODS/STUDY POPULATION: The study population includes children with medical complexity (CMC), a medically fragile pediatric population with intensive healthcare needs. CMC rely on multiple and complex medication regimens and/or medical devices for optimal functioning. Parents of CMC report multiple unmet healthcare needs. For Aim 1, we will conduct observations and interviews with ˜15 clinicians as well as semi-structured interviews with ˜30 family caregivers during three care transition experiences: from Cardiac ICU to home, Neonatal ICU to home, and those between primary care/specialty clinic to home. For Aim 2, we will conduct participatory design sessions with up to 5 participants (separately for clinicians and family caregivers) from each of the three care transition settings to co-design a prototype intervention. RESULTS/ANTICIPATED RESULTS: The study is currently recruiting family caregivers of CMC for aim 1 research activities, with interviews planned to be completed in February/March 2021. Transcribed interviews will be used to inform development of patient journey maps. A patient journey map helps to visually depict healthcare services through the patient and family lens, and highlights important ‘touch points’ along the patient journey (e.g., decisions, encounters, constraints, emotional states, etc.) that shape the patient and family experience. The journey map will distill findings from qualitative data and generate a concise visual story focused on the medication use experience of CMC as they transition between the hospital and their home. Individual journey maps will also be combined to generate a consolidated journey map. DISCUSSION/SIGNIFICANCE OF FINDINGS: An-in-depth understanding of medication safety risks unique to the context of CMC care would be essential to develop interventions that are useful, scalable, and sustainable. This is even more important because current interventions are primarily adopted from adult care settings with mixed outcomes.

